# Transcriptome Sequencing and WGCNA Reveal Key Genes in Response to Leaf Blight in Poplar

**DOI:** 10.3390/ijms241210047

**Published:** 2023-06-12

**Authors:** Ruiqi Wang, Yuting Wang, Wenjing Yao, Wengong Ge, Tingbo Jiang, Boru Zhou

**Affiliations:** 1 State Key Laboratory of Tree Genetics and Breeding, Northeast Forestry University, Harbin 150040, China; yjswrq@outlook.com (R.W.); wyt1513026188@163.com (Y.W.); yaowenjing@njfu.edu.cn (W.Y.); 2 Co-Innovation Center for Sustainable Forestry in Southern China, Bamboo Research Institute, Nanjing Forestry University, Nanjing 210037, China; gavingo@njfu.edu.cn

**Keywords:** WGCNA, leaf blight, poplar, SOD, POD, co-expression network

## Abstract

Leaf blight is a fungal disease that mainly affects the growth and development of leaves in plants. To investigate the molecular mechanisms of leaf blight defense in poplar, we performed RNA-Seq and enzyme activity assays on the *Populus simonii × Populus nigra* leaves inoculated with *Alternaria alternate* fungus. Through weighted gene co-expression network analysis (WGCNA), we obtained co-expression gene modules significantly associated with SOD and POD activities, containing 183 and 275 genes, respectively. We then constructed a co-expression network of poplar genes related to leaf blight resistance based on weight values. Additionally, we identified hub transcription factors (TFs) and structural genes in the network. The network was dominated by 15 TFs, and four out of them, including *ATWRKY75*, *ANAC062*, *ATMYB23* and *ATEBP*, had high connectivity in the network, which might play important functions in leaf blight defense. In addition, GO enrichment analysis revealed a total of 44 structural genes involved in biotic stress, resistance, cell wall and immune-related biological processes in the network. Among them, there were 16 highly linked structural genes in the central part, which may be directly involved in poplar resistance to leaf blight. The study explores key genes associated with leaf blight defense in poplar, which further gains an understanding of the molecular mechanisms of biotic stress response in plants.

## 1. Introduction

Leaf blight is a prevalent fungal disease that primarily affects plant leaves. The disease typically starts from leaf margins and tips and gradually spreads from local to systemic, causing the leaves to wither and eventually fall off. Severe infections can even lead to plant death. To defend against pathogens, plants have evolved a sophisticated defense system that is typically categorized into two main branches by the type of pathogen molecules they recognize: pattern-triggered immunity (PTI) and effector-triggered immunity (ETI) [1,2,3]. Generally, the PTI can be activated by pathogen-associated molecular patterns (PAMPs) through transcription of immunity-related genes, resulting in the accumulation of reactive oxygen species (ROS) and secondary metabolites and the activation of mitogen-activated protein kinase (MAPK) cascades and Ca^2+^ signals [4,5,6]. ROS accumulation is easy and convenient to detect quantitatively. Additionally, SOD (Superoxide Dismutase) and POD (Peroxidase) activities can represent the ability of plants to scavenge ROS, which is usually regarded as a measure of plant susceptibility to response to adversity stress. SOD is a key enzyme that resists biological oxidation in plants by catalyzing the reduction of superoxide anions (O^2−^) to hydrogen peroxide (H_2_O_2_). The diminished capacity of O^2−^ removal decreased the ability of progeria cells to minimize oxidative damage, which plays a critical role in the defense of cells against the toxic effects of oxygen radicals [7,8]. POD constitutes a class of enzymes widely distributed in plants. An important function of POD is involved in lignin synthesis, which provides cell wall rigidity and allows the plant to protect itself. In many cases, especially for plant–microbe interactions, this is considered to be a defense strategy for plants under stress conditions [9].

Transcriptome sequencing technology allows the profiling of gene expression patterns related to plant–pathogen interactions, which enriches the functions of related genes and proteins. For example, an epoxide hydrolase, which was induced by the fungal pathogen *Alternaria alternata* on rough lemon, was isolated via transcriptome sequencing [10]. Transcriptome sequencing revealed the resistance genes affecting the expression of specific *Aspergillus flavus* genes in *Zea mays* [11]. Similar studies have also been reported in apples [12], tomato [13,14], *Nicotiana benthamiana* [15] and other plant species. However, little is known about the initial exploration of regulatory mechanisms of leaf blight defense in poplar via transcriptome sequencing. Previous studies indicated that *PsnWRKY70*-overexpressing transgenic plants displayed enhanced resistance to *A. alternata* in poplar by activating the genes in both pathogen-associated molecular pattern-triggered immunity (PTI) and effector-triggered immunity (ETI) [16]. In the study of *Populus davidiana* × *Populus bollena* against leaf blight, *A. alternata* was found to induce the differential expression of defense-related genes and ROS metabolism-related genes, and the hub gene *PdbLOX2* could enhance the resistance to *A. alternate* in transgenic plants [17].

Weighted gene co-expression network analysis (WGCNA), which is capable of extracting associated genes from phenotypic data, is widely used for exploring the complex relationships among different kinds of genes [18,19,20,21,22,23]. In this study, we sampled the poplar leaves with inoculation of *Alternaria alternate* fungus for 0 h, 12 h, 24 h, 48 h and 72 h, respectively. Additionally, we performed RNA-Seq and enzyme activity assays of the samples and applied WGCNA technology to correlate enzyme activity and gene expression patterns, to extract the genes directly related to leaf blight defense, and finally constructed co-expression networks to reveal the core transcription factors (TFs) and structural genes. In this study, we initially revealed the key gene networks of poplar resistance to leaf blight, which sheds light on the understanding of the molecular mechanisms of biotic stress response in plants.

## 2. Results

### 2.1. Changes in SOD and POD Activities in Poplar Leaves with Inoculation of Leaf Blight Fungus

**Figure 1:**
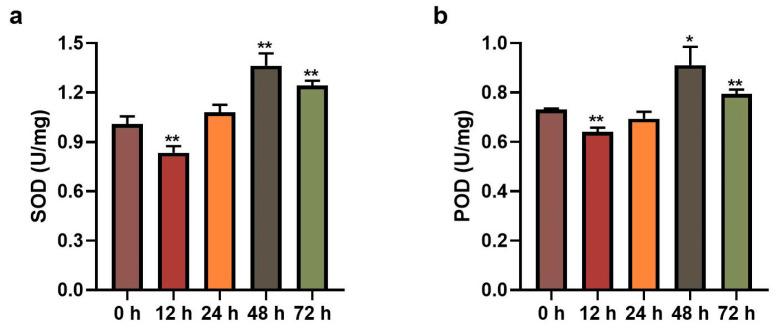
Detection of (**a**) SOD and (**b**) POD activities in poplar leaves with inoculation of leaf blight fungus. Error lines represent the standard deviation of three biological replicates, and asterisks represent significant differences (*t*-test, * *p* < 0.05, ** *p* < 0.01).

To investigate the effects of leaf blight fungus on poplar leaves, we measured the activities of SOD and POD in poplar leaves inoculated with *Alternaria alternate* fungus for 0 h, 12 h, 24 h, 48 h and 72 h, respectively. As shown in Figure 1 and Table S1, the expression trends of SOD and POD activities were similar at different time points, which decreased first and then increased. The activities of both enzymes were significantly down-regulated after 12 h of fungus inoculation and significantly up-regulated after 48 h. The consistency of the two enzyme activities indicates that poplar leaves displayed an obvious response to leaf blight fungus.

### 2.2. Transcriptome Sequencing

A total of 43,294,816, 40,140,892, 46,301,748, 44,057,436 and 44,989,982 raw reads were obtained from the RNA-Seq of the samples with the treatment of *Alternaria alternate* fungus for 0 h, 12 h, 24 h, 48 h and 72 h, respectively. Additionally, respective 37,547,669, 34,230,032, 40,135,884, 33,991,073 and 29,407,893 clean reads were obtained after removing the low-quality reads (Table S2). A total of 70.54~95.40% of the clean reads could be mapped to the reference genome of *Populus trichocarpa*. (FPKM values were calculated based on the counts of the mapped unigenes (Table S3). A total of 32,384 genes with functional annotations were identified via Nr annotation, accounting for 78.34% of all of the obtained genes (Table S3). Overall, the genes have high homology with the reference genome and can be analyzed subsequently.

### 2.3. WGCNA Reveals Gene Modules Associated with Poplar Leaf Blight

**Figure 2:**
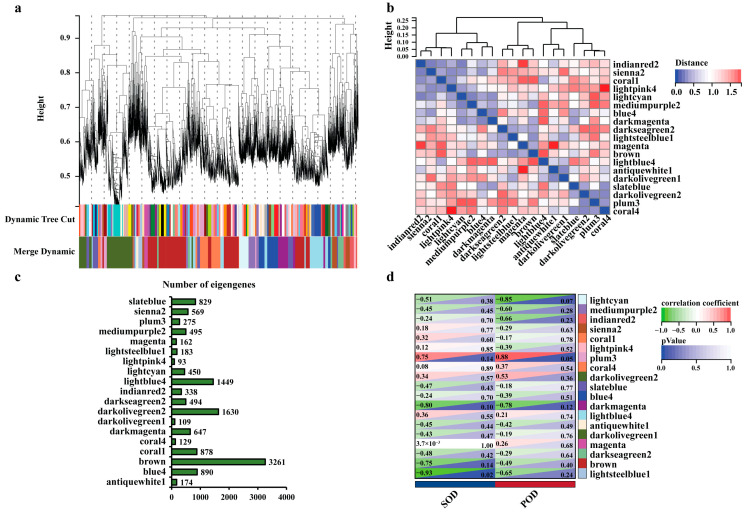
WGCNA reveals the modules associated with poplar leaf blight. (**a**) Clustering dendrogram of genes, with dissimilarity based on topological overlap, together with assigned module colors. (**b**) Heatmap showing the Pearson correlation among the eigengenes in co-expressed gene modules. (**c**) Statistics on the number of eigengenes in different modules. (**d**) Heatmap of the correlation between different modules with POD and SOD. The correlation coefficients were colored according to the score.

To identify the genes associated with leaf blight, we performed WGCNA analysis on the expression of 41,335 genes in the poplar leaves with incubation of leaf blight fungus for different times. To eliminate the noise from the unexpressed or low-expressed genes, we filtered the probes for median transcripts with FPKM < 1, and set the lower limit to 50 in the module and the sensitivity of module formation to 3. Those genes were grouped into 19 co-expressed modules with their pairwise correlation evaluation (Figure 2a,b, Table S4). Each set of highly correlated genes corresponded to a branch of the tree (Figure 2a). There was often a high topological overlap between genes in the same module. Those 19 modules could be gathered into two clusters that had a high degree of interaction connectivity (Figure 2b). Among all of the modules, the three modules containing the highest number of eigengenes were the brown module (3261), the darkolivegreen2 module (1630) and the lightblue4 module (1449). The three modules with the lowest number of eigengenes were the lightpink4 module (93), the darkolivegreen1 module (109) and the coral4 module (129) (Figure 2c). To identify the co-expression modules that are highly correlated with leaf blight, we evaluated the POD and SOD activity values of poplar leaves with the 19 modules for correlation. The results showed that the module significantly correlated with SOD was lightsteelblue1 with a correlation coefficient of −0.93, and the module significantly correlated with POD was plum3 with a correlation coefficient of 0.88 (Figure 2d). It indicates that the eigengenes in these two modules are most likely involved in leaf blight resistance in poplar.

### 2.4. Expression Pattern and GO Enrichment Analysis of the Eigengenes in Leaf Blight-Related Modules

**Figure 3:**
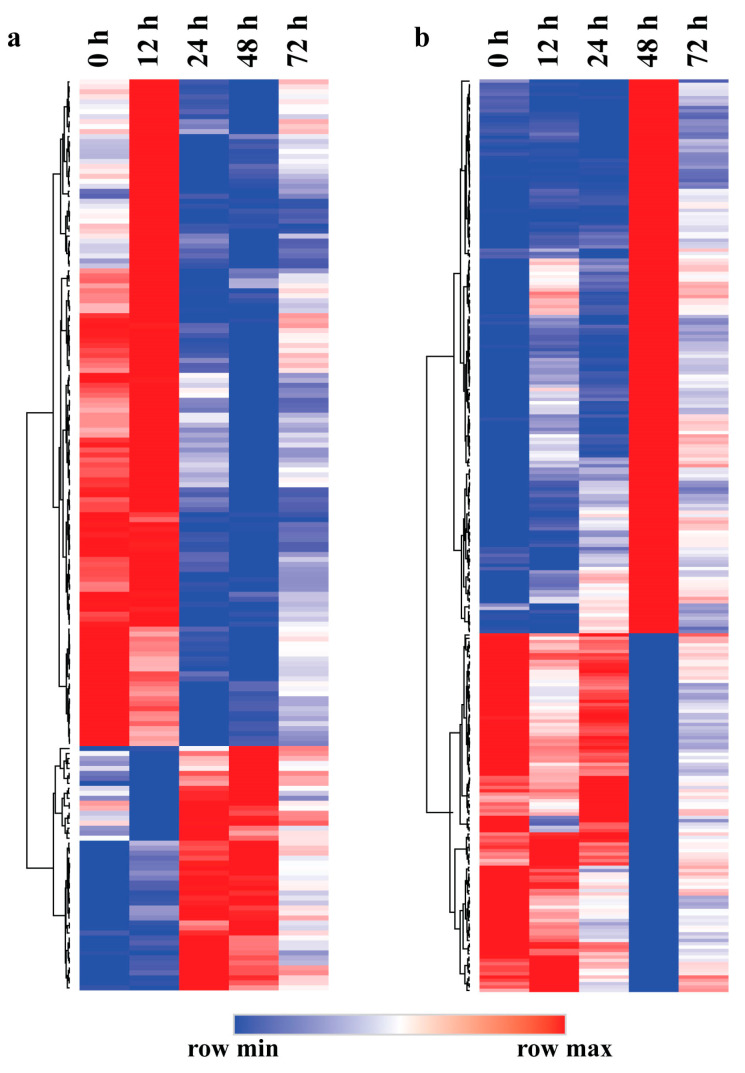
Expression patterns of the eigengenes in different modules related to leaf blight in poplar. (**a**) Eigengenes expression pattern in the lightsteelblue1 module. (**b**) Eigengenes expression pattern in the plum3 module.

To elucidate the functions of the eigengenes in the lightsteelblue1 and plum3 modules, we first performed a clustering analysis of the genes in both modules. As shown in Figure 3a, the eigengenes in the lightsteelblue1 module can be divided into two parts. One part was highly expressed at 0 h and 12 h and lowly expressed at 24–72 h. The other part had the opposite expression pattern, with low expression at 0 h and 12 h and high expression at 24–72 h. It indicates that the expression of the eigengenes in the lightsteelblue1 module showed significant changes after 24 h inoculation of leaf blight fungus. Clustering analysis showed that the genes in the plum3 module were also divided into two parts (Figure 3b). One part was lowly expressed at 0 h, 12 h, 24 h and 72 h and highly expressed at 48 h. The other part had the opposite expression pattern, with high expression at 0 h, 12 h, 24 h and 72 h and low expression at 48 h. It showed that the eigengenes in the plum3 module responded to leaf blight fungus obviously after 48 h of inoculation.

**Figure 4:**
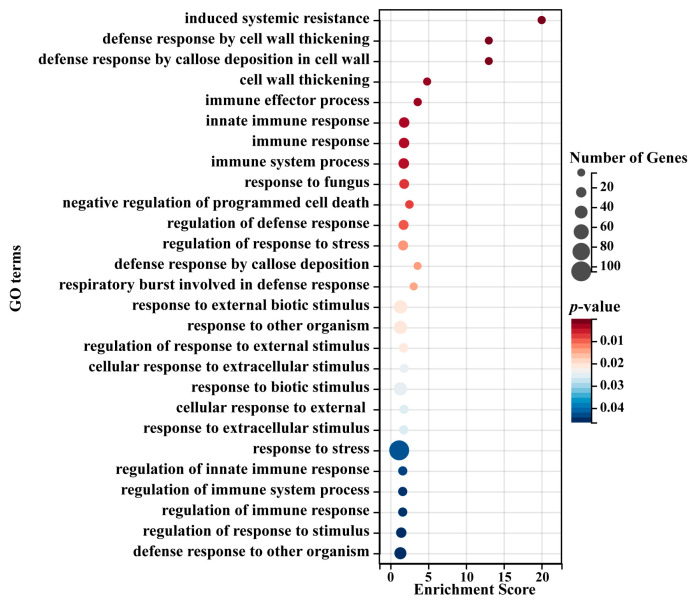
Typical significantly enriched GO terms for the eigengenes in the leaf blight-related modules. The x-axis represents an enriched enrichment score. The y-axis represents GO terms. The size of each circle represents the number of genes, and color represents *p*-value. *p*-value ≤ 0.05 indicates significant enrichment.

We then performed GO enrichment analysis of all leaf blight-related eigengenes in both modules. The results showed that a total of 141 significantly enriched GO terms were obtained, including 116 biological-process-related terms, 8 cellular-component-related terms and 17 molecular-function-related terms (Table S5). In the biological process category, the three most abundant significantly enriched terms were response to stress (GO:0006950), response to chemical (GO:0042221) and response to organic substance (GO:0010033). In the cellular component category, the three most abundant significantly enriched terms were cell periphery (GO:0071944), plasma membrane (GO:0005886) and endomembrane system (GO:0012505). In the molecular function category, the three most abundant significantly enriched terms were oxidoreductase activity (GO:0016491), electron transfer activity (GO:0009055) and tetrapyrrole binding (GO:0046906). In addition, in the biological process category, we found several GO terms related to plant resistance to leaf blight, including response to stress (GO:0006950), response to external biotic stimulus (GO:0043207), response to other organisms (GO:0051707), response to biotic stimulus (GO:0009607), defense response to other organisms (GO:0098542), and so on (Figure 4). Overall, the eigengenes in both modules are directly related to plant biotic stress-related biological processes, suggesting that these genes play an important function in poplar resistance to leaf blight.

### 2.5. Functional Analysis of Co-Expression Network of the Modules Related to Leaf Blight in Poplar

**Figure 5:**
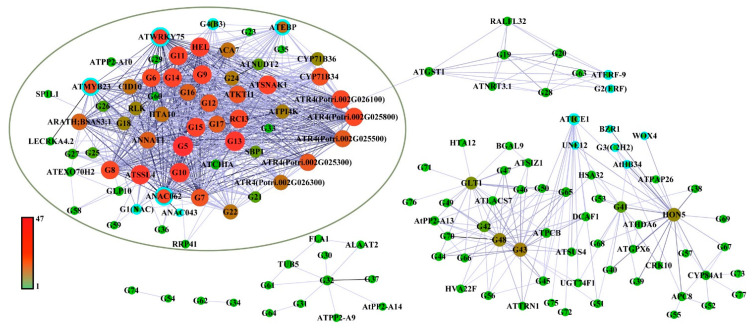
Co-expression network of the genes related to leaf blight in poplar. Node size and coloring depth represent node connectivity. Nodes with blue borders represent transcription factors. The information on G1–G77 is listed in Table S7. The coloring line depth represents the Weight value. The green oval area indicates highly interacting gene clusters.

To obtain the key genes in the co-expression network, we filtered the eigengenes pairs in the two leaf-blight-related modules, with weight ≥ 0.4 as the threshold. A total of 957 linear pairs were obtained (Table S6) and subsequently imported into Cytoscape 3.9.1 software for visualization. As shown in Figure 5 and Table S7, the co-expression network contains a total of 15 TFs and 129 structural genes. These TFs are mainly from NAC (three genes), ERF (three genes) and bHLH (two genes) families. Based on previous reports, multiple homologs of these TFs were found to be involved in plant biotic stress and stress resistance processes [24,25,26,27,28,29,30,31]. Furthermore, the results showed that the network contains a large gene cluster containing a total of 63 genes, 39 of which had a connectivity of more than 20 (Figure 5, Table S8), implying that this gene cluster may be a core regulatory network of poplar resistance to leaf blight. In particular, this gene cluster was dominated by four highly concatenated TFs (concatenation ≥ 30), namely *ATWRKY75*, *ANAC062*, *ATMYB23* and *ATEBP*, which are likely to act as key switches in the regulatory network of poplar resistance to leaf blight.

**Figure 6:**
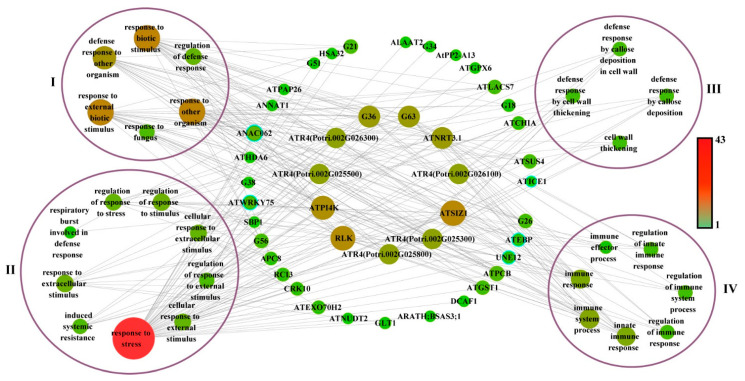
Gene networks in GO terms associated with leaf blight in poplar. Node size and coloring depth represent node connectivity. Nodes with blue borders represent transcription factors. The information on G1–G77 is listed in Table S7. I–IV denote the four GO terms, groups of biotic stress, resistance to stress, cell wall and immune response, respectively.

To further elucidate the function of the co-expression network, we captured the structural genes from the co-expression network based on leaf-blight-related GO terms. A total of 44 structural genes were captured from the network and subsequently linked to GO terms (Figure 6). We classified the GO terms associated with leaf blight into four categories, namely biotic stress, resistance, cell wall and immune response. As shown in Figure 6, there were 16 highly linked structural genes in the central part, including *ATPI4K*, *RLK*, *ATSIZ1*, *ATNRT3.1*, G63 (Potri.018G007300), G36 (Potri.007G062700), *ATR4* (Potri.002G026300, Potri.002G025500, Potri.002G025800, Potri.002G025300 and Potri.002G026100), indicating that these genes are enriched in biological processes highly associated with leaf blight in poplar.

### 2.6. qRT-PCR Validation of Hub Genes in co-Expression Network of Leaf Blight

**Figure 7:**
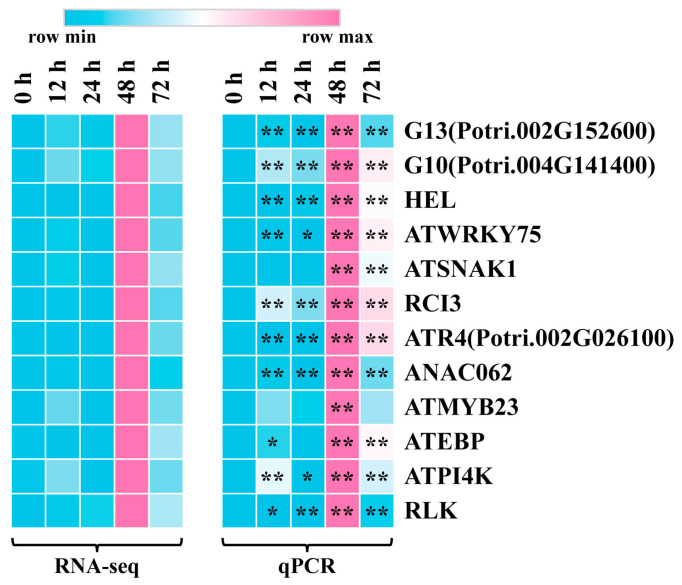
qRT-PCR validation of 12 hub genes. Heatmap on the left is from RNA-seq and heatmap on the right is from qRT-PCR. Data were processed using the 2^−ΔΔCt^ method. Gene expression in 0 h was set to 1 and expression in the other time points was relative to it. The qPCR results were calculated using 3 biological replicates. (*t*-test, * *p* < 0.05, ** *p* < 0.01).

To ensure the accuracy of the expression pattern of the eigengenes associated with leaf blight via RNA-Seq, we selected 12 hub genes with high connectivity in the co-expression network for qRT-PCR validation. As shown in Figure 7 and Table S9, the qRT-PCR results were highly consistent with the RNA-seq results. All of the 12 genes reached extremely significant up-regulated expression levels at 48 h after fungus infestation, which also echoes the results of SOD and POD activity assays.

## 3. Discussion

### 3.1. WGCNA Reveals the Gene Modules Directly Related to Leaf Blight in Poplar

POD and SOD are two main enzymes in the antioxidant system of plants, and their activity levels can reflect the extent to which plants are affected by external adversity. In the study, after poplar leaves were inoculated with leaf blight fungus, we detected significant changes in the activities of POD and SOD, indicating that the poplar was activated by biotic stress. We then performed RNA-Seq on poplar leaves inoculated with leaf blight fungus and correlated gene expression levels with SOD and POD enzyme activity levels to obtain the core modules (lightsteelblue1 and plum3) associated with leaf blight via WGCNA. With the clustering analysis of the expression levels of the eigengenes in the two modules, we found that the overall expression pattern of the SOD-related module lightsteelblue1 showed a remarkable change after 24 h in leaf blight-infested plants, indicating that these genes were activated/repressed to function after 24 h treatment. In contrast, the expressions of the eigengenes in the POD-related module plum3 were characterized by significant changes at 48 h, indicating that these genes function after 48 h of fungus infestation. In addition, with GO enrichment analysis of the eigengenes in both modules, we identified several significant enrichment biological processes associated with plant resistance to leaf blight, such as response to stress, response to external biotic stimulus, response to other organisms, response to biotic stimulus, defense response to other organisms, and so on. The overall indication is that the genes contained in the two modules revealed via WGCNA are associated with plant resistance to leaf blight.

### 3.2. Co-Expression Network and Function Analysis of the Genes Related to Leaf Blight in Poplar

In general, a hierarchical transcriptional regulatory network is the main operational strategy to classify plant genes involved in growth and development and stress response. Additionally, the network is generally dominated by many TFs, which act as the network hub [32,33,34,35,36]. In the study, we constructed a co-expression network of the genes directly related to leaf blight in poplar by filtering the weight value between the eigengenes in the lightsteelblue1 and plum3 modules. A total of 15 TFs were covered, among which four TFs had high connectivity in the network. Some of these 15 TFs have been reported to be related to biotic stress in other plant species, such as the overexpression of *AtWRK75*-induced oxidative burst in host plants, which suppressed the hyphal growth of *Sclerotinia sclerotiorum* and consequently inhibited fungal infection [24]. *PsnWRKY70* gene has been reported to enhance leaf blight resistance of transgenic poplar lines [37]. *ERF9* participates in the plant defense process against necrotic fungi mediated by the DEAR1-dependent ethylene/JA signaling pathway [27]. *AtEBP* expression can be induced by gray mold infection, and the interaction of ACBP4 and AtEBP may be related to AtEBP-mediated defense [28]. The BZR1-EDS1 module may be harnessed for the simultaneous improvement of crop productivity and pathogen resistance [30,31]. There are also some TFs in our network that have been reported to be involved in abiotic stress responses. For example, *AtICE1* confers multiple stress tolerance in indica rice, and the role of ICE1 in stress tolerance and stomatal development is conserved across plant species [29]. *ANAC062* was reported to be associated with stress resistance in Arabidopsis [26]. Similarly, the remaining unreported TFs, such as *AtHB34*, *WOX4*, G1 (Potri.005G098000, *NAC* family), *ANAC043*, *ATMYB23*, G2 (Potri.006G138700, *ERF* family), G3 (Potri.009G043900, *C2H2* family), *UNE12*, and G4 (Potri.015G126500, *B3* family), may play important functions in poplar resistance to leaf blight.

For the structural genes in highly interacting gene clusters in the network, some have also been reported to be associated with stress resistance, such as the *RLK* gene involved in the LRR-RLK machinery mediating immune responses in Arabidopsis [38]. In poplar, *LRR-RLK* was also found to be involved in leaf blight resistance, which was regulated by PsnWRKY70 [16]. The *HEL* gene is a defense gene in plants that is associated with disease resistance in plants [39,40]. *SSL4* and *SSL5-7* play specific roles in plant defense mechanisms [41]. *AtKTI1* is involved in modulating PCD in plant–pathogen interactions. The RNAi silencing of *AtKTI1* leads to enhanced resistance to the virulent pathogen *Erwinia carotovora* subsp [42]. *ANNAT1* has been reported to be involved in abiotic stress responses and plant immune responses [43,44]. In addition, the GO term–genes network constructed in the study covers a large number of potentially functionally important structural genes with high connectivity in the co-expression network, such as *RLK*, *ATPI4K*, *ATR4* and so on. These structural genes have been annotated in the GO enrichment analysis to be involved in several biological processes related to biological stress, resistance to stress, cell wall and the immune system. It indicates that these co-expression networks contain structural genes that may also play a role in leaf blight resistance under the activation/repression of their upstream TFs in poplar.

## 4. Materials and Methods

### 4.1. Plant Materials

*P. simonii × P. nigra* is a kind of hybrid poplar widely distributed in northern China (from the Heilongjiang River to the Yellow River), which has advantages in terms of rapid growth and strong adaptability to saline, cold, drought and barren conditions. Thus, the poplar species is usually considered to be an ideal woody plant for the study of stress responses [37,45,46]. In this study, di-haploid *Populus simonii × Populus nigra* seedlings were grown at 26 °C with a 16 h light/8 h dark cycle in the experimental field of Northeast Forestry University (Harbin, China). Two-month-old seedlings were sprayed with prepared *Alternaria alternata* spore suspension (1.0 × 107 spores mL^−1^), as previously described [47,48,49]. The third to eighth layer of functional leaves were harvested at 0 h, 12 h, 24 h, 48 h and 72 h, respectively. To guarantee the stability of the test of RNA-seq results, each sample was prepared by mixing leaves derived from the same location of 18 plants. The sampled materials were immediately frozen in liquid nitrogen and stored at −80 °C for enzyme activity assay and RNA-Seq.

### 4.2. SOD and POD Activity Analysis

The activity of SOD and POD was measured using SOD and POD assay kits (COMIN, Suzhou, China) according to the instructions for the procedure, respectively.

### 4.3. Transcriptome Sequencing

The sampled poplar leaves were sent with dry ice to GENEWIZ company (Suzhou, China) for high-throughput Illumina sequencing (Illumina, San Diego, CA, USA). After the high-throughput output of a large amount of high-quality raw data, most of its bases can be scored at or above Q30. To ensure that the raw reads are of sufficiently high quality, low-quality reads, such as the reads containing connectors, were all removed to obtain clean reads. Additionally, the obtained clean reads were mapped to the *Populus trichocarpa* reference genome (https://phytozome-next.jgi.doe.gov/ (11 June 2020)) using HISAT 2 software [50]. Then, the mapped reads were assembled and quantified using StringTie [51]. The FPKM values of the genes were subsequently calculated using RSEM [52].

### 4.4. Weighted Gene Co-Expression Network Analysis

We constructed the co-expression networks using WGCNA in the R package [53], which is a scale-free network construction method that identifies gene clusters with highly correlated expression profiles. We estimated the Pearson correlation coefficients among the genes based on their FPKM values by converting the correlation matrix into an adjacency matrix. Hierarchical clustering and dynamic tree cut function were used to detect modules, grouping all genes into clusters. For high reliability of the results, the minimum number of genes was set to 50, and the sensitivity was set to 3.0. Gene significance (GS) and module membership (MM) were calculated to correlate the modules with the phenotypic data (SOD and POD enzyme activities). The information of the corresponding module genes was extracted for further analysis.

### 4.5. Gene Annotation and GO Enrichment Analysis

Gene annotation was conducted using the Nr annotation tool in Omicshare tools (https://www.omicshare.com/, accessed on 1 Marth 2023). GO enrichment was performed using a free online data analysis platform, Pop’s Pipes [54]. Additionally, GO enrichment analysis was represented as three separate hierarchies of molecular function, biological process and cellular component, with *p*-value ≤ 0.05 indicating significant enrichment. The results were visualized using TBtools [55].

### 4.6. qRT-PCR Analysis

Three samples were prepared for RT-qPCR at each time point (each sample was identical to the one used for RNA-seq). The total RNA (isolated from RNA-Seq samples) was used to synthesize first-strand cDNA using the Hifair^®^ miRNA 1 st Strand cDNA Synthesis Kit (Yeasen, Shanghai, China). The Hieff^®^ qPCR SYBR^®^ Green Master Mix (Yeasen, China) was applied to identify the gene expression patterns, and *PtActin* was used as am endogenous reference gene. Primers are shown in the Table S10. The 2^−ΔΔCT^ relative quantification method was used to analyze the relative changes in gene expression. Standard errors and standard deviations were calculated from three biological replicates.

### 4.7. Data Analysis

Standard errors and standard deviations were calculated using SPSS 21 (Chicago, IL, USA). A *t*-test was used for significance analysis with *p*-value ≤ 0.05 as the statistically significant level. The data were presented as mean ± standard error (SE) from three independent biological samples.

## 5. Conclusions

In the study, we investigated the molecular mechanism of leaf blight defense in poplar leaves by correlating enzyme activity traits with gene expression profiles through the WGCNA approach. Two significantly related eigengenes modules, lightsteelblue1 and plum3, containing 183 and 275 genes, respectively, were obtained. The co-expression network analysis indicated that a total of 15 TFs were hub centers in the network, among which four dominated TFs, including *ATWRKY75*, *ANAC062*, *ATMYB23* and *ATEBP*, which had extremely high connectivity and were in the pivotal position in the network. In addition, a total of 16 structural genes with high connectivity were also identified in the center part of the network, which may be directly involved in poplar resistance to leaf blight. The study revealed that these key genes with high connectivity in the co-expression network play important functions in leaf blight defense in poplar, which further gains an understanding of the molecular mechanisms of biotic stress response in plants.

## Data Availability

The datasets used and analyzed in this study are available in the Sequence Read Archive (SRA) at NCBI at the National Center for Biotechnology Information. The accession number is PRJNA963172.

## Supplementary Materials

The following supporting information can be downloaded at: https://www.mdpi.com/article/10.3390/ijms241210047/s1.
